# An Analysis of New Feature Extraction Methods Based on Machine Learning Methods for Classification Radiological Images

**DOI:** 10.1155/2022/3035426

**Published:** 2022-05-25

**Authors:** Firoozeh Abolhasani Zadeh, Mohammadreza Vazifeh Ardalani, Ali Rezaei Salehi, Roza Jalali Farahani, Mandana Hashemi, Adil Hussein Mohammed

**Affiliations:** ^1^Department of Surgery, Faculty of Medicine, Kerman University of Medical Sciences, Kerman, Iran; ^2^Robotics Research Laboratory, Center of Excellence in Experimental Solid Mechanics and Dynamics, School of Mechanical Engineering, Iran University of Science and Technology, Tehran, Iran; ^3^Industrial Engineering Department, Technical and Engineering Faculty, University of Science and Culture, Tehran, Iran; ^4^Department of Electrical Engineering, Islamic Azad University, Tehran, Iran; ^5^School of Industrial and Information Engineering, Politecnico di Milano University, Milan, Italy; ^6^Department of Communication and Computer Engineering, Faculty of Engineering, Cihan University-Erbil, Erbil, Kurdistan Region, Iraq

## Abstract

The lungs are COVID-19's most important focus, as it induces inflammatory changes in the lungs that can lead to respiratory insufficiency. Reducing the supply of oxygen to human cells negatively impacts humans, and multiorgan failure with a high mortality rate may, in certain circumstances, occur. Radiological pulmonary evaluation is a vital part of patient therapy for the critically ill patient with COVID-19. The evaluation of radiological imagery is a specialized activity that requires a radiologist. Artificial intelligence to display radiological images is one of the essential topics. Using a deep machine learning technique to identify morphological differences in the lungs of COVID-19-infected patients could yield promising results on digital images of chest X-rays. Minor differences in digital images that are not detectable or apparent to the human eye may be detected using computer vision algorithms. This paper uses machine learning methods to diagnose COVID-19 on chest X-rays, and the findings have been very promising. The dataset includes COVID-19-enhanced X-ray images for disease detection using chest X-ray images. The data were gathered from two publicly accessible datasets. The feature extractions are done using the gray level co-occurrence matrix methods. *K*-nearest neighbor, support vector machine, linear discrimination analysis, naïve Bayes, and convolutional neural network methods are used for the classification of patients. According to the findings, convolutional neural networks' efficiency linked to imaging modalities with fewer human involvements outperforms other traditional machine learning approaches.

## 1. Introduction

COVID-19 revealed flaws in many countries' healthcare services, and the failure of these systems to treat patients has generated concern. The lack of accuracy in clinical detection methods is significant for COVID-19's rapid dissemination [1]. Molecular methodologies, such as computational real reverse transcription-polymerase chain reaction (rRT-PCR) [2], as well as other techniques, such as serologic tests [3] and swab testing of the throat [4, 5], are employed and widely utilized to diagnose COVID-19. Researchers also used chest radiographs (X-rays) and chest computed tomography (CT) scans to help indicate abnormalities characteristic of various lung diseases, including COVID-19. CT scans and X-ray examinations may be used as a critical screening method to assess COVID-19 seriousness, track infectious patients' emergency cases, and forecast COVID-19 progression [6, 7]. However, time is always short in such situations. These trials cannot be carried out using the current standard manual diagnosis [8]. Deep learning (DL) is a branch of machine learning (ML) that learns the highest-level abstractions from findings using hierarchical structures. DL is utilized to solve artificial intelligence challenges due to the multiple computational levels. The layers of DL are created from data using a learning process rather than by individual technologists, which is a crucial feature of the DL approach. Its benefits, such as higher achievement, blended function learning with an end-to-end learning scheme, and the potential to monitor composite knowledge challenges, have achieved relevant therapeutic effectiveness in healthcare. DL algorithms are also being employed to track and evaluate coronavirus pandemics, thanks to their extensive application in the research of medical recordings. Some theorists and simulation engineers used autoencoders, convolutional neural networks (CNN), and generative adversarial networks to analyze the coronavirus pandemic. Mathematical and predictive methods may estimate human loss and predict death over a specific time frame or up to the end of the pandemic. Since statistical simulations do not consider all facets of the pandemic, they cannot make more precise predictions. To construct powerful models and resources that support clinicians detect COVID-19 infection, scientists and modeling engineers have widely used intelligent computation methods in healthcare. The government is taking the necessary measures to prohibit the COVID-19 pandemic from spreading and advancing intelligent computing methods in healthcare. As a result, several ML approaches are being used to tackle the latest COVID-19 pandemic successfully. de Moraes Batista et al. [9] proposed that various machine learning methods are used for the prognosis of novel coronaviruses. Moreover, training the network with 70% reference data and 30% test data measured the accuracy of different ML techniques. With 68% sensitivity, 85% specificities, 85% AUC, and 0.16 Brier scores, the support vector machine (SVM) outperforms random forests, logistic regression, and gradient boosting tree techniques. ML approaches have several drawbacks, including the heavy use of patient data [10], inconsistency, dependence on temporal data, paucity, discrepancy [11], and the failure to produce accurate forecasts due to high dimensionality [12]. The generalized logistic growth model was utilized by Ahmadi et al. [13] to predict the subepidemic waves of the COVID-19 outbreak. Two, three, and four-wave phenomena were predicted using the formula. Centered on a lung X-ray map, Hassantabar et al. used an ML algorithm to detect infected COVID-19 patient tissue [14, 15]. This discovery can also be utilized to observe and manage patients' progress in contaminated areas [16–20].

This paper employs machine learning methods to classify COVID-19 X-ray images. The feature extraction process is the first and most crucial step in the classification process in machine learning. Even though many deep learning algorithms employ input photos to train the network, the GLCM feature extraction approach shown in this study is successful. As opposed to featuring extraction techniques with an image input, the essential point in feature extraction methods with picture input is to lower the processing time. The size of the output network is significantly less than with image input. The last phase involves the application of machine learning technologies and the extraction of network performance data. The plans are compared in the confusion matrix and ROC curve. For the classification of patients, KNN, SVM, LDA, NB, and CNN methods are used.

## 2. Literature Review

By learning from basic depictions, deep learning strategies can clarify complicated problems. DL methods have become popular because they learn exact representations and the property of studying information in a fundamental method where several layers are used sequentially [21]. DL techniques are usually employed in medical science, biomedical science [20, 22], innovative health [23, 24], drug delivery [25], and medical image recognition [26, 27], among others. It is also commonly used to make an automated COVID-19 diagnosis. Data analysis, data processing, feature extraction and classification, and performance assessment are all stages in deep learning-based systems [21, 28]. A pretrained method has already been trained in fields related to the application's context. Weight and prejudice were retrained in transfer learning from a limited training network to a new study network. In particular, training big data takes a long time and needs much computing capital [21]. Xu et al. evaluated a novel approach employing multitask joint training algorithm for sectioning and classifying tongue images using a deep convolutional neural network [29].

In another research by using support vector collection data, a multiple kernel-based fuzzy SVM model was developed to predict DNA-binding proteins by Zou et al. [30]. Using a pretrained transfer learning model, the facility speeds up the convergence with network generalization [31]. In transfer learning, multiple pretraining networks are required for the huge CNN. Some of the pretrained network of COVID-19 classifications include AlexNet [32], GoogleNet [33], SqueezeNet [34], various variants of visual geometry group (VGG) [35], various forms of ResNet [36], Xception [37], various forms of inception [38], various types of MobileNet [39], DenseNet [40], U-Net [41, 42], and others. Using transfer learning, detecting 0 in CT and X-ray images has been effectively extended. 3D CT images are handled distinctly other than colored X-ray. 3D CT images have a set number of slices based on the computer and configuration (16, 32, 59, 128, etc.). Individual slices of nature may be greyscale or color photographs. Usually, the slices are isolated and treated as individual pictures [43]. The slices with the most lung areas are included, while the remaining slices are removed. Rezaei et al. created a lithological cartography in the Sangan region of Northeastern Iran using satellite data and image processing technologies [44]. During COVID-19, Arenliu et al. have statistically analyzed the building of online and telephone psychological first aid services in a low-resource setting [45]. Rezaei et al. introduced a data-driven approach for segmenting hand parts on depth maps that did not need any additional effort to acquire segmentation labels [46]. In [47], case study features obtained from the slices are utilized to optimize the pretrained network. Also, U-Net has been utilized to segment and reduce features from various regions of interest (ROI) in 3D CT in some cases [48]. Rehman and Lela investigated how to manage crises during the COVID-19 pandemic [50]. Radulescu and Cavanagh adapted their SEIR standard model [51] to investigate the complicated hierarchical compartments and epidemiologic factors of COVID-19. They reviewed the new management policies of the pandemic, mutual isolation, travel prohibition, interruptions, and closings, to produce predictions and assess the feasibility of containment measures. The aim of this study was to estimate the distribution gap in COVID-19 by means of a hybrid of SEIR models and regression models applying data from the John Hopkins University on COVID-19 [52]. Sadeghipour et al. conducted different research in which they compared the Expert Clinical System for Diagnosing Obstructive Sleep Apnea to the XCSR classifier [54]. According to the study by Zhang et al., a privacy-preserving algorithm is implemented for querying clinical pathways in e-healthcare systems [55]. Liu et al. published a self-supervised CycleGAN method for super-resolving ultrasound images with perception consistency [56]. Several ways to reduce the negative economic effects of COVID-19 were presented by Mahmoudi et al. [57]. Chaudhary et al. proposed the Fourier-Bessel series expansion-based decomposition technique, a Fourier-Bessel series expansion domain application of the wavelet packet decomposition methodology. Using a transfer learning technique, the subband pictures are utilized for training multiple pretrained CNN models independently. A feature set is obtained by fusing the in-depth features of each channel. Several classifiers distinguish pneumonia caused by COVID-19 from other viral and bacterial pneumonia and healthy patients with the recovered feature vector. The COVID-19 categorization based on chest X-rays requires a radiology specialist and a substantial amount of time, both valuable commodities when COVID-19 illness spreads rapidly. As a result, creating an automated analytic technique is desirable to save time for medical experts. The research discussed in [59] uses the susceptible exposed infected removed (SEIR) method for physical estimation and evaluation distancing. If the process of return to work started in April, physical distance measures were successful. This study investigates the relationship between temperature and humidity in relation to the transmission of COVID-19 [60]. Alyasseri et al. [57] divided the research tracks into two categories: deep learning (DL) and machine learning (ML), and they presented COVID-19 public datasets that had been produced and retrieved from different nations. The measurements used to evaluate diagnostic approaches have been compared, and a thorough explanation has been provided. The study served as a guide for the research community on the planned development of machine learning for COVID-19. It served as an inspiration for their work on subsequent developments.

Kumar et al. [76] investigated various artificial intelligence-based strategies to apply these techniques to the prediction and diagnosis of COVID-19 illness. Their ideas for future study and their facilitation of knowledge collecting and formulation on the application of artificial intelligence approaches for dealing with the COVID-19 outbreak and its effects were much appreciated. Using computed CT scans, Akram et al. [77] demonstrated an automated approach for the quick diagnosis of COVID-19 infection. They used discrete wavelet transform and extended segmentation-based fractal texture analysis methods to extract the essential characteristics from the fractal texture dataset. Khan et al. [78] presented a strategy for enhancing contrast by combining top-hat and Wiener filters, which they called “contrast enhancement.” Two deep learning models that had already been trained were used and fine-tuned according to the target classes. The features were then retrieved and fused using a parallel fusion approach—the parallel positive correlation—to achieve the best results. The entropy-controlled firefly optimization approach was used to identify the most optimal characteristics. Nejatishahidin et al. [79] proposed a new pose estimation method that can be applied to previously undetected environments. A framework for identifying 15 different forms of chest illnesses, including the COVID-19 disease, was suggested by Rehman et al. [80]. The framework was based on the use of a chest X-ray modality. This architecture enhanced the accuracy of COVID-19 identification while simultaneously increasing the prediction rates for other chest disorders. According to one suggestion, face masks can be used nationwide and applied promptly (see Table 1).

## 3. Methods and Materials

### 3.1. Feature Extraction

The extraction feature decreases the number of tools used to interpret large data sets accurately. The vast number of variables involved is one of the critical problems of sophisticated data analytics. It would help if you had plenty of memory and computing power for working with an extensive range of variables or required an algorithm to classify the exhibits and use them for new samples. Feature extraction is a wide-ranging concept to create dynamic mixes of variables to tackle these problems with absolute accuracy. Texture analysis sought to identify a reliable way to represent the specific properties of textures in a simplified but specific way for correct object detection and division. The surface is essential in image analysis and pattern recognition. Texture extraction is only used in a few processor architectures [75].

A gray surface incidence matrix is developed in this study to achieve statistical texture characteristics. The texture properties of the observed substances are calculated from the statistical light intensity distribution of specific positions in the statistical texture analysis concerning each other. The numbers are classified as first, second, or higher order, depending on the number of pixels for each combination. The gray level matrix (GLCM) is a technique to calculate statistical texture characteristics of the second order. In several recent studies, this method has been used for third and higher-order textures that recognize the relationships between three or more physically feasible pixels but are seldom used because time and perception complexity are computed. GLCM has 22 features, which are listed as follows [81]:

1. Energy
2. Entropy
3. Dissimilarity
4. Contrast/inertia
5. Inverse difference
6. Correlation
7. Homogeneity
8. Autocorrelation
9. Cluster shade
10. Cluster prominence
11. Maximum probability
12. Sum of squares
13. Sum of average
14. Sum of variance
15. Sum of entropy
16. Difference variance
17. Difference entropy
18. Information correlation criteria (1)
19. Information correlation criteria (2)
20. Maximum correlation coefficient
21. Inverse difference normalized
22. Inverse difference moment normalized

### 3.2. Machine Learning Classifiers

One of the learning networks inspired by the perceptron neural network is this kind of neural network. The picture or features linked to the problem are first classified and fed into the grid. The concealed weights in the output layer would then express themselves in several ways [61]. If the output comprises multiple numerical components, the algorithm gives a classification or recognition algorithm (e.g., image classification, normal = 1, abnormal = 2). After many images have been trained, the results are weighted.

The image form is detected when a new image is applied to the algorithm, other than the training images. For instance, the matrix of different images is sent to the method and trained in the computer, such as images of benign or malignant cancers, Alzheimer's, sarcoma, or brain tumors [61]. The process determines the type of disease based on the weights obtained. The convolutional sublayer is the CNN's foundation, and its output matrix can be seen as a 3D matrix of neurons. Standard neural networks are considered for a better understanding. Each layer was a 1D matrix of neurons, producing its output and eventually accumulating a series of results corresponding to each neuron. While rather than a scaler number, a 3D matrix in which the neurons are arranged in 3D in the CNN is revealed. As a result, this cube's output is also a three-dimensional matrix [61]. The placement of a maxpooling layer among several layers is a widespread technique in traditional architecture. This layer aims to reduce the features and measurements in the network and thus overfit the display, resulting in a smaller input size. The maxpooling feature enlarges or reduces the size of the position. Artificial neural networks' activation mechanism specifies the neuron based on the input matrix [82]. The result values are converted to a goal range, 0 to 1 or −1 to 1 (depending on the activation mechanism used). For instance, the logistic activation function converts all inputs to true absolute ranges [0, 1]. Another way to lose weight is to optimize your weight. In this article, the rectified linear unit (ReLU) for the following functions is employed:

(1) $$\begin{array}{c} f\left(x\right)=\left\{\begin{array}{cc} 0 & x<\text{0,}\\ \text{x} & x\geq 0. \end{array}\right. \end{array}$$

The expressions, such as SoftMax, are not exclusive to a single feature and relate to the 1D matrix.

(2) $$\begin{array}{c} {\text{f}}_{\text{i}}\left(\overset{⟶}{x}\right)=\begin{array}{c} e^{x_{i}}\\ \sum_{j=1}^{J}e^{x_{j}} \end{array}. \end{array}$$

In KNN, each tuple in the n-dimensional space can be called a point if the educational tuples contain *n* indexes. The *k*-nearest algorithm is specified based on distance measures (e.g., Euclidean distance) in each attribute when a nontuple is given. The test sample is found among the training samples. The test sample's classmark is identical to the labels of the majority of these samples in the test sample's vicinity [83].

SVM is a supervised ML technological category that uses a hyperplane to classify each observation in a given data set. SVM can address linear and nonlinear issues, which is more beneficial in large datasets. SVMs are a generalized linear classifier that can be considered a perceptron extension. They are also known as a particular case of Tikhonov's regularization [84].

Bayesian learning is a computational method for linking data sets to different mathematical approaches by learning conditional independence. Bayesian learning incorporates previous probability functions and new insight to measure later probability. The probability of (*θ*) must be amplified if *Y*_1_, *Y*_2_, *Y*_3_,…*Y*_*n*_ represents a set of inputs and returns a label. Naive Bayes classifiers can be trained effectively for particular probability models in a supervised learning framework. The maximum likelihood approach is used to estimate parameters for naive Bayes models in many practical applications. In other words, the naive Bayes model [85] can be used without endorsing Bayesian probability or using complex Bayesian techniques.

Fisher's linear discriminant is a technique for determining a linear combination of variables that describe or discriminate two or more sets of objects or occurrences. It is utilized in statistics and other areas. Fisher's linear discriminant is a generalization of LDA. The resulting mixture may be used as a linear classifier or, more generally, reduce dimensionality before further classification. Discriminant analysis is used when groups are known a priori (unlike in cluster analysis). A score on one or more quantitative predictor variables, as well as a score on a group indicator, is required for each scenario [86]. In basic terms, discriminant function analysis involves grouping, classifying, or categorizing objects into similar groups, classes, or categories.

### 3.3. Classifier's Performance Analysis Metrics

The ROC curve is defined by comparing the true positive rate (TPR) to the false positive rate (FPR). In ML, the TPR is also called recall or probability of detection. Starting on the ROC's left side, the FPR and TPR have vanished. (This implies that the threshold line, which represents the most significant number of test results, is extensive.) Start with the most test results and use that as a starting point. The consistency of the findings of a measure that divides the knowledge into these two categories is measurable and descriptive.

  True positive (TP): the classification of the COVID-19 patient is accurate
  False positive (FP): the classification of non-COVID patients is with mistakes
  True negative (TN): the classification of non-COVID is accurate
  False negative (FN): the classification of COVID-19 patients is with mistakes

In mathematical terminology, the sensitivity of separating the percentages of TP cases into the number of TP and FN cases is as follows:

(3) $$\begin{array}{c} \text{Sensitivity}=\frac{\text{TP}}{\text{TP}+\text{FN}}. \end{array}$$

**Figure 1:**
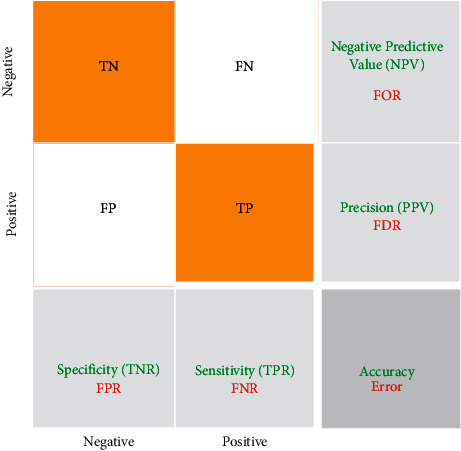
The confusion matrix.

The function of the techniques listed in artificial intelligence is the confusion matrix. Such a presentation is often utilized in supervised learning algorithms, but it is often employed in unsupervised learning. Each column of the matrix contains an instance of the expected value. Suppose each row has an actual (true) case. Also, the name of this matrix is revealed, allowing for errors and tampering with the outcome [86] (see Figure 1).

## 4. Results and Discussion

### 4.1. Dataset

**Figure 2:**
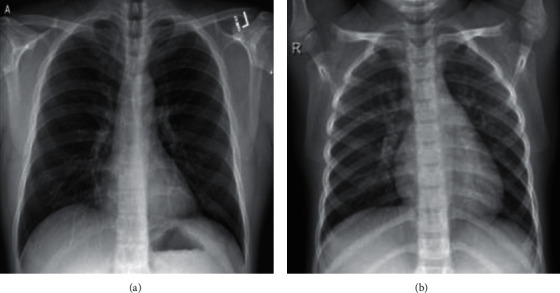
Example of X-ray image from patients' lungs. (a) COVID-19 patient. (b) Other patients.

The dataset includes COVID-19-enhanced X-ray images for disease detection using chest X-ray images. The data were gathered from two publicly accessible datasets [87, 88]. The data are based on a shared open dataset of chest X-rays and CT photographs of patients with COVID-19 or other viral or bacterial pneumonia and are positive or suspicious (MERS, SARS, and ARDS). Data would be compiled directly from hospitals and doctors and indirectly from public records. All photographs and data are made public [46, 50, 53]. Images are in the size of 256 × 256 in PNG format. The example of images is shown in Figure 2. For analysis and simulation of the methods, 1824 image is used so that 80% belongs to training and 20% is the validation sample. [89].

### 4.2. Preprocessing

**Figure 3:**
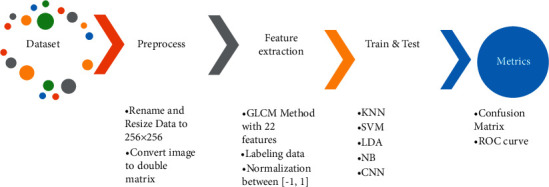
Conceptual diagram of the presented method.

In this paper, the dataset of lung X-ray images is collected from the data repository. It converted to a unique PNG format with 256 × 256 pixel size. First, the image should be transformed into a double matrix for analysis. The first and most crucial part of classification in machine learning is feature extraction. Although many deep learning methods use input images to train the network, this paper's GLCM feature extraction method is effective. The critical point in feature extraction methods is that the processing time is reduced, and the size of the output network is highly more minor than the methods with image input. The GLCM approaches extracted 22 features for each image explained in the methods and materials section. The features are normalized between [−1, 1] in the next step to transform the matrix in an identical range. Two folders of COVID-19 patients and non-COVID patients with 1824 images were finally converted to a matrix with an 1824 × 22 size. Then, images with COVID-19 infections are labeled with (1 or positive) and other images (0 or negative). The final step is the implementation of machine learning methods and extracting performance metrics from the networks. The conceptual diagram of the process is illustrated in Figure 3.

### 4.3. Results of Classification

In this paper, five powerful machine learning techniques are utilized to classify and diagnose the COVID-19 patients with other patients. Five classifiers that analyze and implement diagnosis include KNN, SVM, NB, LDA, and CNN.

**Figure 4:**
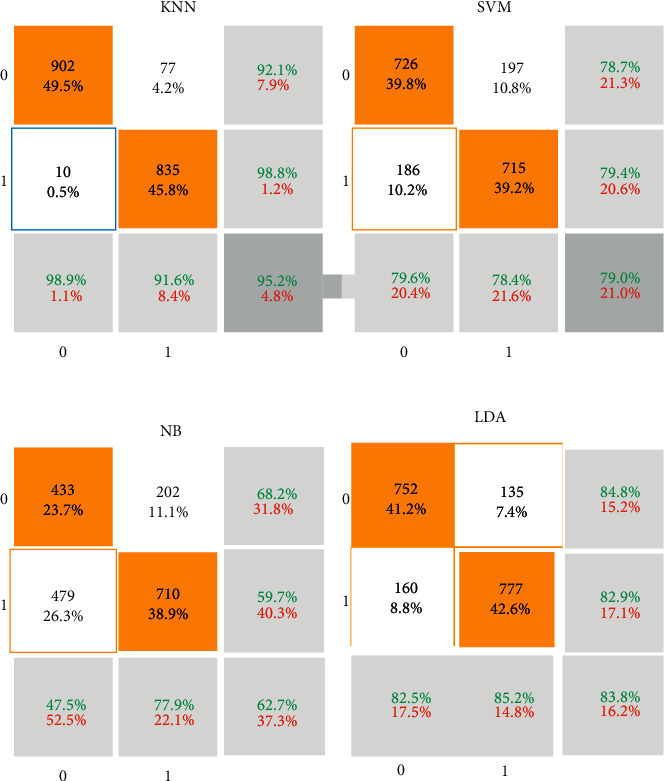
The confusion matrix of the deep learning methods used for COVID-19 diagnosis.

Moreover, each class of 912 vectors with 22 features enters the methods. The classification results in a confusion matrix are depicted in Figure 4. Regarding the confusion matrix in Figure 4, orange cells illustrate true metrics, and white cells are false. For example, in KNN results, from 912 images with COVID-19, 825 (45.8% of all images) are detected correctly. However, 77 (4.2% of all images) are misdiagnosed. Therefore, the sensitivity of the KNN methods is 91.6%. In other words, 91.6% of patients with COVID-19 are detected accurately.

Moreover, from 912 images with other diseases, 98.9% are diagnosed correctly. It means that the specificity of the KNN is 98.9%. Furthermore, precision for KNN is 98.8%, which means that 845 patients are detected as COVID-19; however, 835 patients (98.8%) are diagnosed truly. Finally, another important metric for analyzing classifiers is accuracy. It includes all number of true values over the number of true and false values. For the KNN method, the accuracy is 95.2%. It means that from all images, 95.2% (902 + 835) of them are diagnosed correctly. Compared with other machine learning methods, the accuracy of KNN is higher than SVM, NB, and LDA approaches. CNN is a feedforward neural network that trains to remove more complex, high-level functionality as neural networks to generate an output map. The convolutional kernel utilizes an input function map. If a function converts, the output map reflects it, so CNN takes advantage of the fact that a feature is the same in the receptive field no matter where it is. It suggests that CNN can learn more helpful functionality than approaches that do not consider. Because of this assumption, weight sharing is used to minimize the number of factors. The CNNs learn through gradient descent. Each layer feeds into the one below it, resulting in hierarchical features tailored to the task at hand. Features in the form of a real-valued vector are typically required by SVM and others.

**Figure 5:**
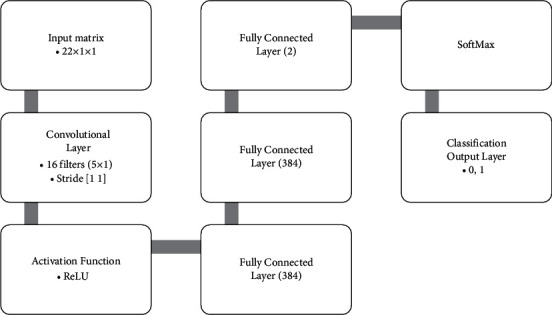
The architecture of presented CNN methods for features classification.

On the other hand, CNN is typically taught from beginning to end, ensuring it responds to the dilemma it is trying to solve. SVMs, KNNs, and random forests use CNN as a trainable attribute detector. Different algorithms in machine learning should be complementary since SVMs are still widely used; it only depends on the task. Therefore, this paper uses the CNN method to classify COVID-19 patients using GLCM features. The structure of CNN architecture is shown in Figure 5.

**Figure 6:**
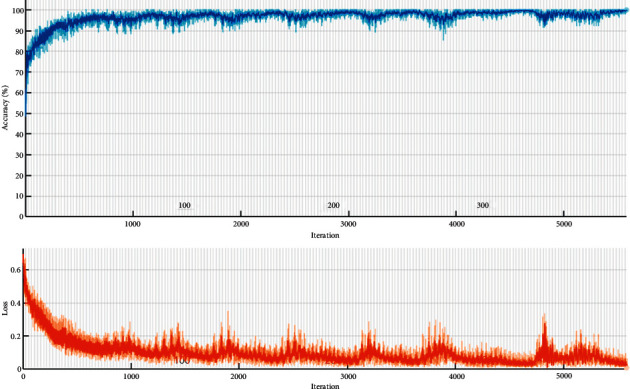
The training process of the CNN approach.

The input layer consists of 22 GLCM normalized features. It is used as a vector for the simulation of the network. Finally, the categorical output labels are 1 for positive samples (COVID-19 patients) and 0 for negative samples (other patients). The simulation has PC core i7, 2.5 GHz, and 12 GB RAM. The process of training is shown in Figure 6. The process has continued to 5800 iterations to reach an acceptable accuracy. The complexity of SVM is *O*(*n*^3^), KNN *O*(*n*  *dk*), NB *O*(*n*  *d*), LDA *O*(*mn*.min(*m*, *n*)), CNN *O*(*n*).

**Figure 7:**
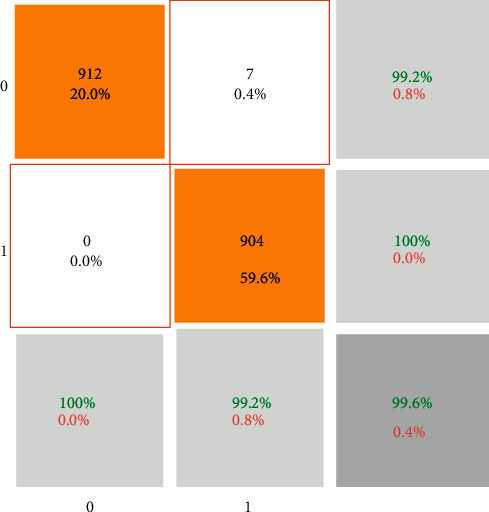
The confusion matrix of the presented CNN method.

The confusion matrix of the CNN results is illustrated in Figure 7. Based on the matrix, from 912 COVID-19 patients, 905 (99.2%) were diagnosed correctly. However, only seven images are misdiagnosed. On the other hand, all the patients with other diseases or negative results are detected. In other words, the sensitivity and specificity of the CNN methods are 99.2% and 100%. Moreover, the precision is 100%. It means that all the patients detected as COVID-19 are truly correct.

**Figure 8:**
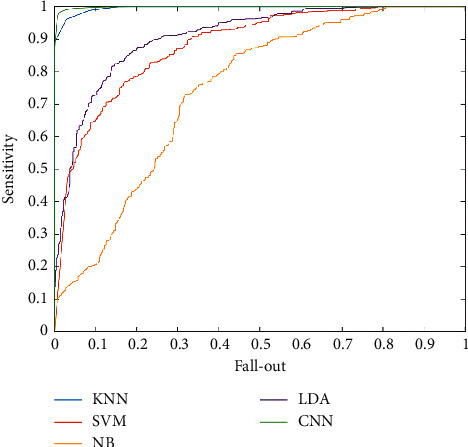
The ROC curve for presented methods.

Consequently, the accuracy of the CNN methods is 99.6%. For comparison of the used machine learning methods, the ROC curve is depicted in Figure 8. Regarding the ROC curve, the horizontal axis is fallout or FPR, and the vertical axis is sensitivity or TPR. If the fallout and sensitivity are lower and higher values, respectively, it is desirable for classification. Therefore, CNN methods result in higher sensitivity in comparison with other methods. Also, NB illustrated lower sensitivity between machine learning methods.

Based on the performance analysis metrics results shown in Table 2, the higher values belong to the CNN technique. Under the ROC curve value, AUC is another essential metric for classifiers. For CNN methods, it is 99.97. Consequently, the highest accuracy belongs to CNN, KNN, LDA, SVM, and NB, respectively.

## 5. Discussion

As medical image processing technology has progressed, intelligent detection and diagnosis software have accelerated. A vital tool for boosting diagnosis accuracy is machine learning algorithms, widely regarded as adequate. However, to generate superior machine learning models, it is vital to use effective feature extraction methods. Therefore, deep learning models are often utilized in medical imaging applications because they can automatically retrieve features or employ pretrained models. As a consequence, deep learning models are becoming increasingly popular. The findings of this article, which employs machine learning algorithms to identify COVID-19 on chest X-rays, are extremely encouraging in their preliminary nature. GLCM algorithms are used to extract the features from the data.

The followings are the broad thumb guidelines employed in selecting textural features: because the values of energy are within a normalized range, it is chosen over entropy. The average gray level difference between neighboring pixels is connected with contrast. It is similar to variance, but it is chosen because of the lower computing effort and usefulness as a spatial frequency measure. In addition, energy and contrast are essential characteristics in terms of visual evaluation and computational burden to discern between distinct textural patterns. The categorization of patients is accomplished using KNN, SVM, LDA, NB, and CNN algorithms. It would be beneficial to have a large amount of memory and computer capacity for working with many variables or require an algorithm to categorize the exhibits and apply them to fresh samples. Feature extraction is a broad idea that may be used to build dynamic mixes of variables that can solve these challenges with pinpoint precision. To achieve accurate object recognition and division, texture analysis tried to discover a reliable technique to express the distinctive attributes of textures in a simplified yet specific manner for object detection and division. The surface is critical in the interpretation of images and the recognition of patterns. Texture extraction is only employed in a few CPU architectures, and it is not widely available.

Additionally, the accuracy is excellent. It follows that all of the patients who were previously diagnosed as having COVID-19 were correct in their diagnosis. Consequently, the best accuracy is achieved by CNN, KNN, LDA, SVM, and NB, in that order. By categorizing lungs and adding information paired with lungs around noise, there is a significant likelihood of supplying more data, resulting in enhanced outcomes. This disturbance is linked to cables and collected equipment, patients' ages, and gender, allowing photos without lungs to be classified precisely. Due to noise bias, future applications utilizing models without lungs may have an increased risk of mislabeling pictures. To verify that noise is not a cause of bias, more work is needed to segregate diseases diagnosed by the expert radiologist. It is also important to note that the findings reported do not always imply the same performance across all datasets. Principal datasets, for example, originate from European patients; other global patients may have modest data capture differences or diseases, implying that a better categorization utilizing global datasets is required. Separating the datasets by gender will also offer further information on the model's scope, as the soft tissues of the breast might conceal sections of the lungs. It is unclear whether this represents a bias in the model's prediction.

## 6. Conclusion

The growth of intelligent detection and diagnostic software has accelerated as medical image processing has advanced. Machine learning algorithms are commonly recognized as a powerful method for improving disease diagnostic accuracy. However, efficient feature extraction tools are necessary to obtain better machine learning models. As a result, deep learning models are commonly used in medical imaging applications because they can automatically retrieve features or use pretrained models. This paper uses machine learning methods to diagnose COVID-19 on chest X-rays, and the findings have been very promising. The feature extractions are done using GLCM methods. For the classification of patients, KNN, SVM, LDA, NB, and CNN methods are used.

The sensitivity of the KNN methods is 91.6 percent, based on the findings. In other words, 91.6 percent of COVID-19 patients are correctly identified. Furthermore, when comparing the process 912 images to other diseases, 98.9% of the disorder is correctly identified. It means that the KNN has a specificity of 98.9%. Furthermore, KNN has a precision of 98.8%, which indicates that 845 patients are observed as having COVID-19 disorder, but only 835 (98.8%) are diagnosed. It means that 95.2 percent of the images are correctly diagnosed. In contrast to other machine learning techniques, the precision of KNN is better than that of SVM, NB, and LDA. Based on the index, 905 (99.2%) of 912 COVID-19 patients were correctly diagnosed. Only seven images, however, are misdiagnosed. Patients with other diseases or unfavorable effects, on the other hand, are identified. In other words, the CNN approaches have a sensitivity and specificity of 99.2 percent and 100 percent, respectively. Furthermore, the precision is fine. It means that all of the patients who were identified as having COVID-19 are right. As a result, CNN, KNN, LDA, SVM, and NB have the best accuracy, respectively. Future works should extend feature-based methods for classifying diseases such as COVID-19, Alzheimer's, and lung cancer instead of image base method. It prevents time constraints and develops fast methods [90, 91].

## Figures and Tables

**Table 1:** Literature review related to deep learning method for classification of COVID-19.

Approaches	Dataset	Volume	TPR	Acc
COVID-Net [58]	COVIDx test	13800	0.871	0.926
ResNet50; InceptionV3; Inception-ResNetV2 [59]	GitHub	100	—	98%;
				97%;
				87%
COVNet [60]	Proprietary datasets	4356	0.87	
Deep learning with X-ray [61]	Proprietary datasets	448	0.986	0.967
			6	8
COVIDX-Net (VGG19 and DenseNet201) [62]	Proprietary datasets	50	—	0.9
Barstugan et al. [63]	Proprietary datasets	150	—	0.996
				8
ResNet50 and SVM [64]	GitHub, Kaggle, and Open-i	158	97.	0.953
			29%	8
SVM and random forests [65]	Hospital Israelita Albert Einstein in São Paulo	—	0.067	0.847
			7	
MLT and SVM [66]	Montgomery County X-ray Set and COVID Chest X-ray Set and COVID Chest X-ray dataset master	40	0.957	0.974
			6	8
Li et al. [67]	Proprietary	—	0.8	0.87
SMOTE [68]	Chest X-ray images (Pneumonia)1 and COVID-19 public dataset from Italy	5840	0.967	0.966
Probabilistic model [69]	Kaggle benchmark dataset	51	—	0.994
NLR&RDW-SD [70]	Jingzhou Central Hospital	—	0.9	0.857
RF-based model [71]	Proprietary	—	—	0.875
SMOTE [68]	Chest X-ray images (Pneumonia)1 and COVID-19 public dataset from Italy	5840	0.932	0.931
iSARF [72]	3 University Hospitals (Tongji, Shanghai, Fudan)	—	0.907	0.879
SMOTE [68]	Chest X-ray images (Pneumonia)1 and COVID-19 public dataset from Italy	5840	0.947	0.947
Modified U-Net structure [73]	SIRM	110	—	0.79
Attention U-Net with an adversarial critic model [74]	JSRT, Montgomery, and Shenzhen	1047	—	0.96
InfNet and the Semi-Inf-Net [75]	CCOVID-19 CT segmentation and COVID-19 CT/X-ray collection	1600	0.725	—

**Table 2:** The comparison between the presented methods.

Methods	Sensitivity (%)	Specificity (%)	Precision (%)	AUC	Accuracy (%)
KNN	91.6	98.9	98.8	99.61	95.2
SVM	78.4	76.6	79.4	88.20	79.0
NB	77.9	47.5	59.7	74.24	62.7
LDA	85.2	82.5	82.9	90.94	83.8
CNN	99.2	100	100	99.97	99.6
[14]	96.1	99.7	96.9	—	93.2

## Data Availability

Data are available and can be provided over the emails querying directly to the corresponding author (f.abolhasani@kmu.ac.ir).
